# Translation role of circRNAs in cancers

**DOI:** 10.1002/jcla.23866

**Published:** 2021-06-07

**Authors:** Yaqin Lu, Zhe Li, Chen Lin, Jian Zhang, Zhisen Shen

**Affiliations:** ^1^ Ningbo University School of Medicine Ningbo China; ^2^ Li Huili Hospital Affiliated to Ningbo University School Ningbo China

**Keywords:** cancers, circular RNAs, internal ribosome entry sites, N^6^‐methyladenosine, translation

## Abstract

Circular RNAs (circRNAs) constitute a class of covalently closed RNA molecules. With the continuous advancement of high‐throughput sequencing technology and bioinformatics tools, many circRNAs have been identified in various human tissues and cell lines. Notably, recent studies have indicated that some circRNAs have translational functions. Internal ribosome entry sites and the N6‐methyladenosine modification mediate cap‐independent translation. This review describes these two translation mechanisms and verification methods at the molecular level. Databases (including ORF Finder, Pfam, BLASTp, CircRNADb, CircBase, CircPro, CircCode, IRESite, IRESbase) were used to analyze whether circRNAs have the structural characteristic of translation. CircRNA minigene reporter system containing green fluorescent protein (GFP) confirmed the translation potential of circRNAs. Also, we briefly summarize the roles of proteins/peptides encoded by circRNAs (circFBXW7, circFNDC3B, circLgr4, circPPP1R12A, circMAPK1, circβ‐catenin, circGprc5a, circ‐SHPRH, circPINTexon2, circAKT3) that have been verified thus far in human cancers (triple‐negative breast cancer, colon cancer, gastric cancer, hepatocellular carcinoma, bladder cancer, glioblastoma). Those findings suggest circRNAs have a great implication in translation of the human genome.

## Graphical abstract

Mechanism of cap‐independent translation initiated by IRES (eg, circFBXW7 in glioblastoma) and by m^6^A modification in the 5′‐UTR. USP28, ubiquitin‐specific peptidase 28; YTHDC1, YTH domain‐containing 1; METTL3, methyltransferase‐like 3; METTL14, methyltransferase‐like 14; YTHDF1, YTH domain family protein 1; YTHDF3, YTH domain family protein 3; m^6^A, N^6^‐methyladenosine; eIF4G2, eukaryotic translation initiation factor 4 gamma 2; IRES, internal ribosome entry site; and UTR, untranslated region.

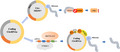

## 1 INTRODUCTION

**FIGURE 1:**
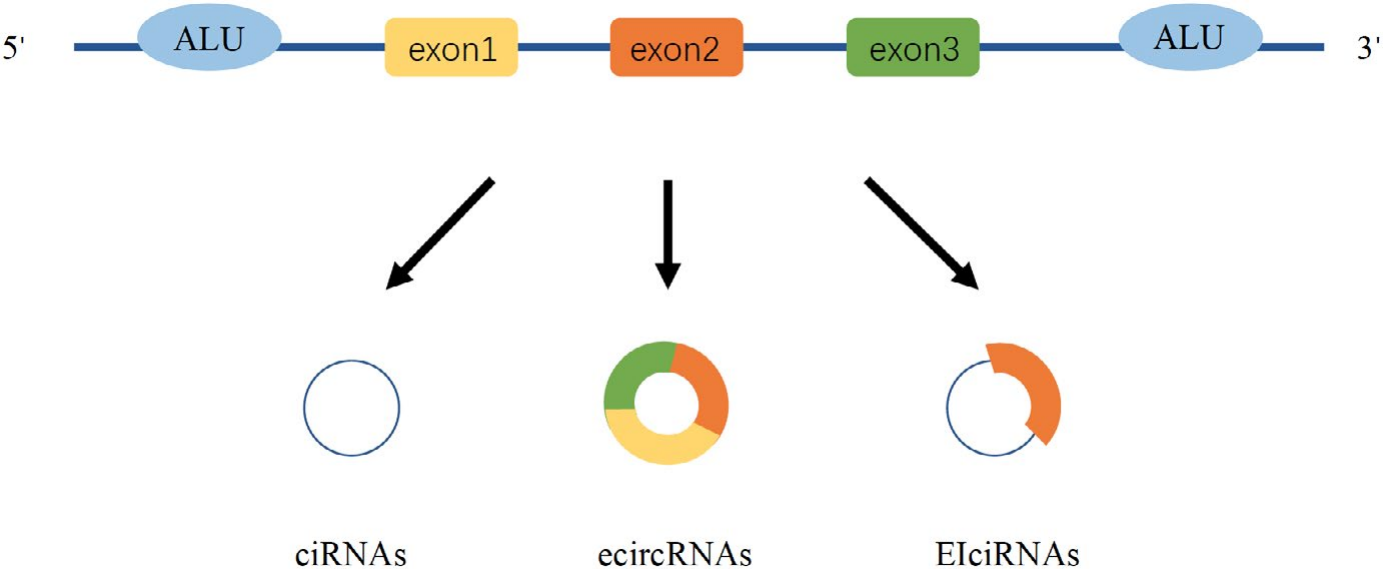
Classification of circRNAs. CircRNAs are divided into circular intronic RNAs (ciRNAs), exonic circRNAs (ecircRNAs), and exon‐intronic circRNAs (EIciRNAs) based on their composition

Circular RNAs (circRNAs) are endogenous RNAs that mostly exist in eukaryotic cells. They are mainly produced by heterogeneous nuclear RNAs (hnRNAs) through the “reverse splicing” mechanism, base pairs in inverted repeat elements (such as ALU), or dimerized RNA‐binding proteins (RBPs) at flanking introns.^1^, ^2^ The downstream splice donor site and the upstream splice acceptor site are covalently linked to form a loop closed structure without a 5′ end cap and 3′ end polyadenylation tail and no terminal structure. Owing to their highly stable and conservative structure, circRNAs can resist the degradation of ribonuclease. Most circRNAs are derived from protein‐coding genes and classified into three categories: intronic circRNAs (ciRNAs), exonic circRNAs (ecircRNAs), or exonic‐intronic circRNAs (EIciRNAs)^3^, ^4^ (Figure 1).

Moreover, the distribution of circRNAs is specific, mainly in the cytoplasm,^5^, ^6^ and their expression can be stably detected in serum, saliva, and tissue samples. Additionally, previous experiments have confirmed that circRNAs are differentially expressed in diseases.^7^, ^8^ Most of these circRNAs have been proposed to have sponging functions and indirectly play a role in promoting/inhibiting tumorigenesis.^9^ Previously, circRNAs were classified as noncoding RNAs due to their highly conserved structure. However, recent studies have shown two important mechanisms critical for regulating the translation function of circRNAs.^10^, ^11^, ^12^ One involves internal ribosome entry sites (IRESs),^13^ and the other involves the N^6^‐methyladenosine (m^6^A) modification^14^; both are potential mechanisms for cap‐independent translation of circRNAs. In other words, cap‐independent translation of circRNAs greatly expands our understanding of the biological functions of circRNAs and provides new perspectives for cancer treatment.

## 2 CAP‐INDEPENDENT TRANSLATION OF CIRCRNAS

### 2.1 Verification of circRNAs translation

In general, traditional translation can be initiated not only by the initiation codon (AUG), a suitable translation sequence and open reading frames (ORFs), but also by a dissociative 5′ end.^10^ Therefore, novel elements are essential for activating cap‐independent translation. To easily understand the requirements for cap‐independent translation, several databases are listed for reference (Table 1). First, the ORFs with coding potential in circRNAs must be identified. ORF Finder^15^ is a graphical sequence analysis tool that can search for all possible ORFs and deduce the translated amino acid sequence. This amino acid sequence is entered into BLASTp or Pfam tools^16^ to confirm that the original search result was successful. Second, a comprehensive circRNA database that can better evaluate the coding potential of circRNAs is accessed. CircRNADb^17^ includes circRNA genome sequence, IRES, and ORF information. CircBase^18^ is constructed through the collection and integration of published circRNA data, which can quickly return circRNA information and supporting evidence. CircPro^19^ can also be used to predict and identify circRNAs with coding potential. CircCode^20^ recognizes translatable circRNAs in ribose sequence reads (ribo‐seq). Finally, IRES is identified. IRESite^21^ contains many published and verified IRESs and provides experimental evidence of these IRESs. IRESbase^22^ includes IRESs related to circRNAs and long noncoding RNAs (lncRNAs), and the function of an IRES has been experimentally verified.

**TABLE 1:** Databases for verifying proteins/peptides encoded by circRNAs

Database	Website	Introduction
ORF Finder	www.ncbi.nlm.nih.gov/gorf/gorf.html	This tool can find all possible ORFs and deduce the translated amino acid sequence.
Pfam	http://pfam.xfam.org/	This tool is used for a homologous search of a protein sequence.
BLASTp	https://blast.ncbi.nlm.nih.gov/Blast.cgi?PROGRAM=blastp&PAGE_TYPE=BlastSearch&LINK_LOC=blasthome"	This tool is used for comparing protein sequences with protein sequences in databases.
CircRNADb	http://202.195.183.4:8000/circrnadb/circRNADb.php	This tool includes circRNAs genome sequence, IRES and ORF information.
circBase	http://cirbase.org/	This tool includes circRNAs information and supporting evidence.
CircPro	http://bis.zju.edu.cn/CircPro	This tool predicts and identifies circRNAs with coding potential.
CircCode	https://github.com/PSSUN/CircCode	This tool recognizes the translatable circRNAs in ribose sequence reads (ribo‐seq).
IRESite	http://www.iresite.org	This tool contains a large number of published and verified IRES.
IRESbase	http://reprod.njmu.edu.cn/cgi‐bin/iresbase/index.php	This tool includes functional IRES that have been experimentally verified.

In addition to using bioinformatics tools, Yang Y et al used the circRNA minigene reporter system to identify functional translation mediated by IRES and m^6^A.^23^, ^24^ By inserting IRES fragments and fragments containing m^6^A motifs before the green fluorescent protein (GFP) sequence, the proteins/peptides can be evaluated by Western blotting (WB) or mass spectrometry (MS). In addition, ribosome profiling and polysome fractionation can infer the movement of ribosomal codons,^25^, ^26^ further confirming that circRNAs have a translational function. To study the biological function of the proteins/peptides encoded by circRNAs, the circRNA transcript is ectopically or endogenously expressed and knocked down or out, and its influence on gene expression and cell phenotype is evaluated.^1^

### 2.2 CircRNA‐encoded proteins/peptides

#### 2.2.1 Translation mediated by IRESs

**FIGURE 2:**
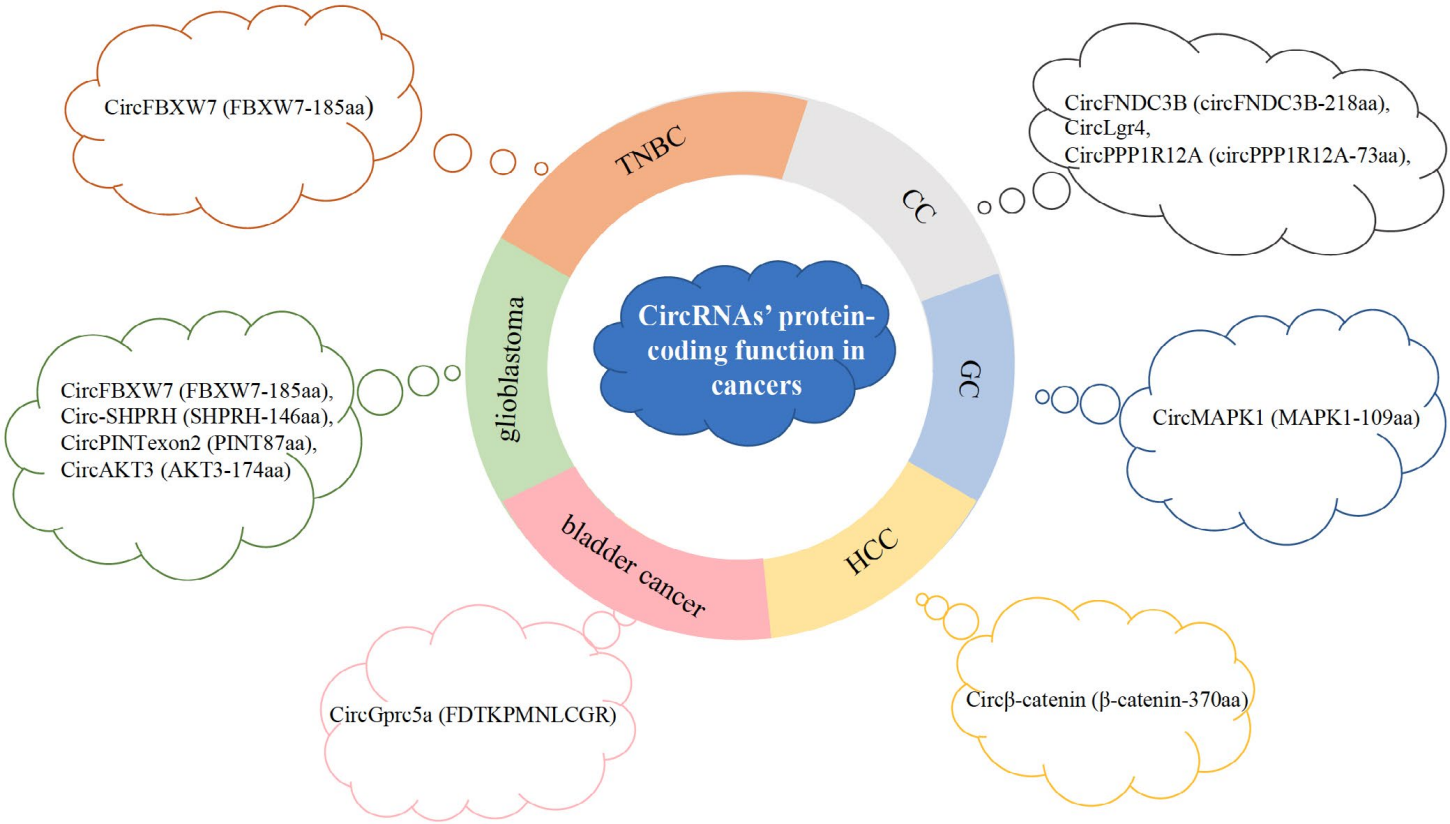
CircRNA‐encoded proteins/peptides in human cancers. CC, colon cancer; GC, gastric cancer; HCC, hepatocellular carcinoma; TNBC, triple‐negative breast cancer

Internal ribosome entry site‐mediated cap‐independent translation is a relatively mature mechanism currently studied. CircRNAs with ORFs and IRESs upstream can be effectively translated by this mechanism in various human cancers (Figure 2).

##### A circRNA‐translated protein in triple‐negative breast cancer (TNBC)

Breast cancer is a common type of malignant tumor and the second leading cause of cancer mortality among women worldwide; TNBC is known as the subtype with the worst prognosis.^27^ Therefore, it is necessary to explore possible mechanisms underlying the progression of TNBC. A study found that circFBXW7 blocks miR‐197‐3p and that the spanning junction ORF of circFBXW7 encodes FBXW7‐185aa,^28^ mediated by IRES, regulates the expression of FBXW7 in TNBC, and exhibits a tumor suppression effect. Therefore, circFBXW7 and FBXW7‐185aa may be potential therapeutic targets.

##### CircRNA‐translated proteins/peptides in colon cancer (CC)

Colon cancer is the second leading cause of cancer‐related death worldwide, and it is a matter of great urgency for researchers to develop more effective molecular targets for CC therapy.^29^ A study reported that circFNDC3B can encode a novel protein, circFNDC3B‐218aa.^30^ More importantly, this protein can perform biological functions independently and inhibit the proliferation, invasion, and migration of CC cells. CircFNDC3B‐218aa attenuates the inhibitory effect of Snail on fructose‐1,6‐bisphosphatase 1 (FBP1) in CC through the Snail‐FBP1‐EMT axis and enhances cell metabolism switching from glycolysis to oxidative phosphorylation, thereby inhibiting the progression of the epithelial mesenchymal transition (EMT) in CC cells. This finding suggests that circFNDC3B‐218aa may be a potential therapeutic target for CC.

The peptide translated by circLgr4 interacts with and activates Lgr4, which further activates the Wnt/β‐catenin signaling pathway, promoting the self‐renewal and tumorigenesis of CC stem cells. Therefore, medicine targeting the circLgr4‐peptide‐Lgr4 axis may be used to treat CC.^31^

Hsa_circ_0000423 (CircPPP1R12A) encodes a peptide, circPPP1R12A‐73aa, which promotes the proliferation, migration, and invasion of CC cells in vivo and in vitro by activating the Hippo‐YAP signaling pathway.^32^ YAP1 is a transcriptional activator in the Hippo signaling pathway, and the transcriptional response induced by YAP1 is crucial to the proliferation and metastasis of cancer cells. YAP‐TEAD inhibitor 1 was used to overexpress circPPP1R12A‐73aa, significantly reducing the proliferation, migration, and invasion ability of circPPP1R12A‐73aa‐expressing CC cells.

##### A circRNA‐translated protein in gastric cancer (GC)

Gastric cancer is the third most common cause of cancer death globally. Due to the lack of specific symptoms of early GC, most patients are diagnosed in the final stage and have poor prognosis. It is urgent to identify biomarkers to improve patient survival. CircMAPK1 (hsa_circ_0004872) encoded a tumor suppressor protein with 109 amino acid length.^33^ MAPK1–109aa inhibits the phosphorylation of MAPK1 by competitively binding MEK1, thereby inhibiting the activation of the MAPK1 pathway and its downstream factors and showing the ability to inhibit the proliferation and invasion of GC cells.

##### A circRNA‐translated protein in hepatocellular carcinoma (HCC)

Hepatocellular carcinoma (HCC) has a high mortality rate, which is attributed to a lack of efficient diagnostic and therapeutic tools.^34^ The Wnt/β‐catenin pathway extensively participates in tumor growth, but the reason it is overactivated in HCC is still unknown.^35^ A recent study showed that circ‐0004194 (circβ‐catenin) encodes a new protein, β‐catenin‐370aa, which is a subtype of β‐catenin.^36^ Additionally, circβ‐catenin regulates the expression of β‐catenin at the protein level, not at the transcription level. β‐catenin‐370aa acts as bait for GSK3β and binds to it, thereby reducing the ubiquitination of β‐catenin by the ubiquitin ligase β‐TrCP, evading the degradation of the proteasome induced by GSK3β. Upregulated β‐catenin enhances the Wnt/β‐catenin pathway and aggravates the malignancy of HCC cells.

##### A circRNA‐translated peptide in bladder cancer

Bladder cancer stem cells exist in the tumor bulk and can induce tumorigenesis. However, the biology of bladder cancer stem cells is unclear.^37^ CircGprc5a is upregulated in bladder cancer stem cells and strengthens the self‐renewal ability of bladder stem cells. In particular, circGprc5a has coding potential and plays a biological role in bladder cancer through the peptide (FDTKPMNLCGR).^38^ It participates in bladder tumor cells by relying on the circGprc5a‐peptide‐Gprc5a axis. Blockers of the circGprc5a‐peptide‐Gprc5a axis may be used for targeted therapy of bladder cancer, which is important because patients whose tumors show high expression of circGprc5a have a poor prognosis.

##### CircRNA‐translated proteins/peptides in glioblastoma

Glioblastoma is one of the most lethal human cancers that may occur at any age and exhibits an extremely poor response to approved therapies.^39^, ^40^ Obviously, new targets/treatments urgently need to be explored. The FBXW7‐185aa protein encoded by circFBXW7 plays a role not only in the aforementioned TNBC,^28^ as mentioned above, but also in glioblastoma.^41^ C‐Myc is a key regulator of tumorigenesis. FBXW7‐185aa competitively interacts with USP28 to prevent the binding of USP28 and FBXW7α and then promotes c‐Myc ubiquitination and degradation. Therefore, FBXW7‐185aa inhibits the proliferation and delays cell cycle progression of glioma cells.

Circ‐SHPRH and its encoded protein SHPRH‐146aa are highly expressed in normal human brains.^42^ Additionally, SHPRH‐146aa protects SHPRH from the degradation of the ubiquitin proteasome and promotes the ubiquitination of proliferating cell nuclear antigen (PCNA), reducing the malignant behavior of cancer cells in vivo and in vitro.

The peptide PINT87aa encoded by circPINTexon2^43^ inhibits the proliferation of glioblastoma cells, and its downregulation induces cell cycle acceleration. Accordingly, the expression of circPINT exon 2 and PINT87a in brain tumor tissues was decreased relative to that in normal brain tissues. It has the same effect in other malignant tumors (including BC, HCC, and gastric cancer), suggesting a poor clinical prognosis. However, a study found that PINT87aa is likely necessary for normal cell survival.^43^

AKT3‐174aa, a tumor suppressor protein encoded by circAKT3, is expressed at low levels in glioblastoma tissues.^44^, ^45^ AKT3‐174aa competitively interacts with phosphorylated PDK1, reduces AKT‐thr308 phosphorylation, and plays a negative regulatory role in modulating the PI3K/AKT signal intensity. Finally, AKT‐thr308 promotes the proliferation and enhances the radiation resistance and tumorigenicity of cancer cells. Furthermore, studies have shown that, in addition to PTEN, AKT‐174aa is also a negative regulator of the RTK/PI3K pathway and may become a potential biomarker for patients with glioblastoma.

#### 2.2.2 Translation mediated by m^6^A modification

CircRNAs modified by m^6^A can also undergo cap‐independent translation.^23^ m^6^A is the most common internal modification of RNAs and has the greatest influence on the regulation of their activity.^46^, ^47^ m^6^A modification is realized by a heterodimeric complex composed of methyltransferase‐like 3 (METTL3), acting as where METTL3 is the catalytic subunit, and methyltransferase‐like 14 (METTL14).^14^, ^48^, ^49^ m^6^a can control pre‐mRNA post‐transcriptional regulation, including splicing, exportation, and translation, by generating conformational changes in local RNA structures or recruiting specific m^6^A reader proteins.^46^, ^50^ The protein containing the YTH domain was the first reader to be recognized. YTHDC1 has been proven to regulate the reverse splicing and export of circRNAs.^51^, ^52^ YTHDF1 was found to increase translation efficiency through the binding of m^6^A and YTHDF3.^53^, ^54^ Then, YTHDF3 and eIF4G_2_ physically associate with endogenous circRNAs to promote the coding of proteins and control cell proliferation. Furthermore, translation from circRNAs is weakened when m^6^A demethylates fat mass and obesity‐associated protein (FTO).^23^, ^55^

A study reported that oncogenic human papillomavirus 16 (HPV16) generates circE7, which translated to produce E7 oncoprotein in CaSki cervical carcinoma cells.^56^ The expression level of E7 oncoprotein affects cancer cell growth both in vitro and tumor xenografts, but the exact carcinogenic mechanism of E7 peptide remains uncertain.

**FIGURE 3:**
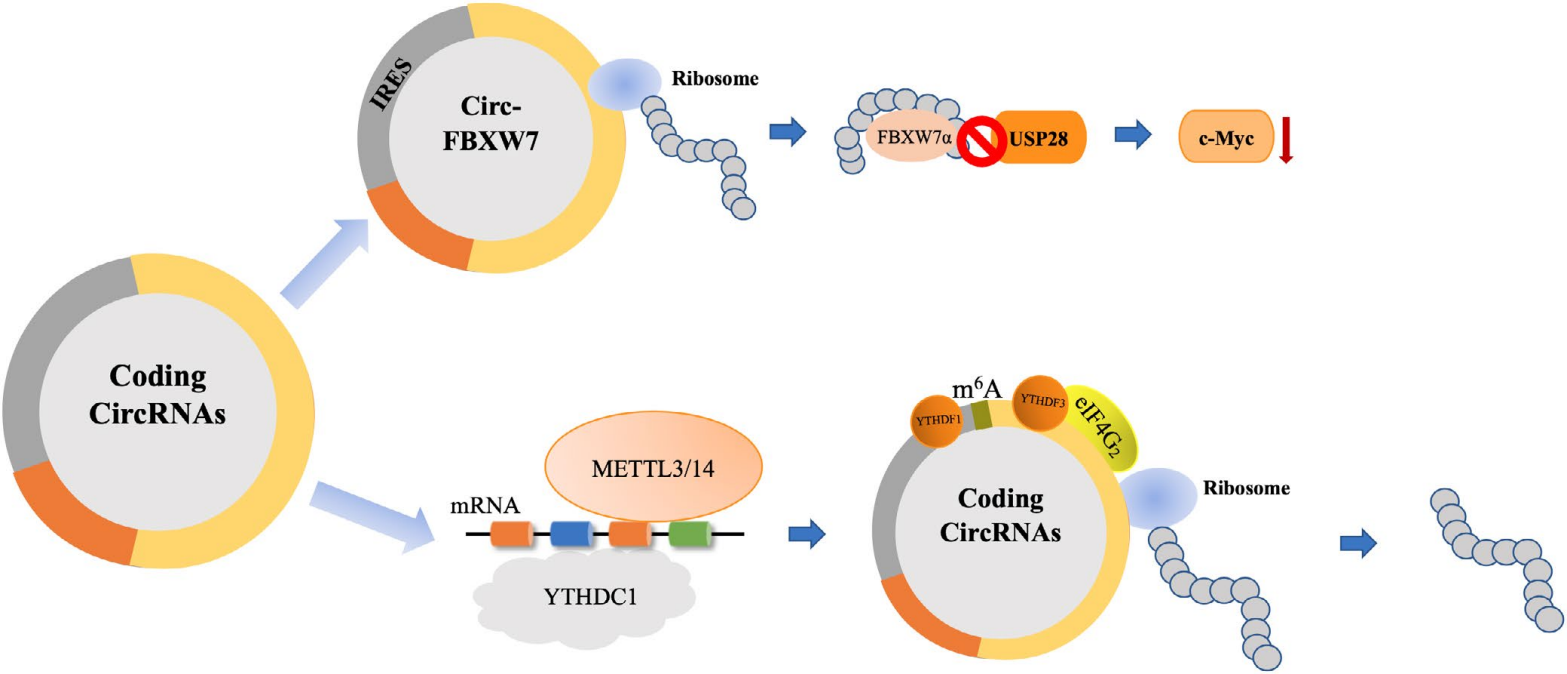
Mechanism of cap‐independent translation initiated by IRES (eg, circFBXW7 in glioblastoma) and by m^6^A modification in the 5′‐UTR. eIF4G2, eukaryotic translation initiation factor 4 gamma 2; IRES, internal ribosome entry site; m^6^A, N^6^‐methyladenosine; METTL14, methyltransferase‐like 14; METTL3, methyltransferase‐like 3; USP28, ubiquitin‐specific peptidase 28; UTR, untranslated region; YTHDC1, YTH domain‐containing 1; YTHDF1, YTH domain family protein 1; YTHDF3, YTH domain family protein 3

In summary, in addition to the IRES‐dependent pathway (Table 2), the m^6^A residues in circRNAs can serve as m^6^A‐induced internal ribosome‐binding sites (MIRES), thereby promoting cap‐independent translation (Figure 3).^14^

**TABLE 2:** The role of circRNA‐encoded proteins/peptides in human cancers

CircRNAs	Protein/Peptide	Cancer	Function	Possible mechanism
circFBXW7	FBXW7‐185aa	Triple‐negative breast cancer	Suppressor	Unknown.
circFNDC3B	circFNDC3B‐218aa	Colon cancer	Suppressor	Inhibit the Snail‐FBP1‐EMT axis.
circLgr4	Peptide	Colon cancer	Promote	Promote the activation of the Wnt/β‐catenin signaling pathway.
circPPP1R12A	circPPP1R12A‐73aa	Colon cancer	Promote	Promote the activation of the Hippo‐YAP signaling pathway.
circMAPK1	MAPK1‐109aa	Gastric cancer	Suppressor	Play a negative regulatory role in the MAPK signal pathway.
circβ‐catenin	β‐catenin‐370aa	Hepatocellular carcinoma	Promote	Enhance the activation of the Wnt/β‐catenin pathway.
circGprc5a	FDTKPMNLCGR	Bladder cancer	Promote	Enhance the activation of the circGprc5a‐peptide‐Gprc5a axis.
circFBXW7	FBXW7‐185aa	Glioblastoma	Suppressor	Increase the expression of FBXW7 and induce c‐Myc ubiquitination‐induced degradation.
circ‐SHPRH	SHPRH‐146aa	Glioblastoma	Suppressor	Promote the ubiquitination of proliferating cell nuclear antigen (PCNA).
circPINTexon2	PINT87aa	Glioblastoma	Suppressor	Interact with the polymerase‐associated factor complex (PAF1c).
circAKT3	AKT3‐174aa	Glioblastoma	Suppressor	Play a negative regulatory role in the PI3K/AKT signal pathway.

## 3 PERSPECTIVES

The biological functions of the proteins/peptides encoded by circRNAs have begun to emerge. We summarized the coding functions of circRNAs in human tumors that have been confirmed thus far and the tumor‐promoting or tumor‐inhibiting effects of their products.

The idea that circZNF609^57^ in myogenesis and circMBL^58^ in fly head extracts can encode peptides initially caused great controversy, but after continuous experimental verification, circRNAs were redefined. In addition, studies have found that some circRNAs have both microRNA (miRNA)‐sponging and protein‐encoding functions and can perform the same, opposite, or irrelevant biological functions in a variety of tissues.^59^ For example, circFBXW7 inhibits malignant progression by sponging miR‐197‐3p and encoding FBXW7‐185aa in TNBC.^60^ Therefore, circRNAs have great potential for use in disease treatment. In the near future, more circRNA‐encoding functions will be verified, and other possible encoding mechanisms will even be discovered.

Although the future of circRNA translation function is very impressive, there are still some problems that need to be considered in depth. For example, the current understanding of the cap‐independent translation mechanism of circRNAs is still unsatisfactory. Are other coding mechanisms possible? The proteins/peptides encoded by circRNAs may serve as new drugs for the treatment of cancers due to their high specificity, low toxicity, and clear biological function. However, their poor stability and short half‐life also greatly limit their clinical application. Therefore, these problems require further research to be solved.

## CONFLICT OF INTERESTS

The authors declare that they have no competing interests.

## Data Availability

The data presented in this study can be found in online repositories. The name of repositories and reference number can be found in the review.
